# Serum CA125 and HE4 levels as predictors for optimal interval surgery and platinum sensitivity after neoadjuvant platinum-based chemotherapy in patients with advanced epithelial ovarian cancer

**DOI:** 10.1186/s13048-016-0270-7

**Published:** 2016-09-27

**Authors:** Aurélie Pelissier, Aurélie Roulot, Béatrice Guéry, Claire Bonneau, Dominique Bellet, Roman Rouzier

**Affiliations:** 1 Department of Breast and Gynecological Surgery, Centre René Huguenin, Institut Curie, 35 rue Dailly, 92210 Saint Cloud, France; 2 Versailles-St-Quentin-en-Yvelines University, EA 7285: Risques cliniques et sécurité en santé des femmes et en santé périnatale, Versailles, France; 3 Laboratory of Biological Oncology, Centre René Huguenin, Institut Curie, 35 rue Dailly, 92210 Saint Cloud, France

**Keywords:** CA125, HE4, Advanced ovarian cancer, Neoadjuvant chemotherapy, Optimal cytoreduction, Platinum sensitivity, Predictive value

## Abstract

**Background:**

The aim of this study is to evaluate a new tumour marker, HE4, and to compare it with CA125 in predicting optimal cytoreduction and response to chemotherapy. Thirty patients with advanced epithelial ovarian cancer and multiple sera harvested during neoadjuvant chemotherapy (NAC) were included.

**Results:**

Based on ROC curves analysis, CA125 ≤ 75 UI/ml and HE4 ≤ 252 pmol/L after the 3rd cycles of NAC, with a sensitivity of 93.7 % and a specificity of 92.3 % (PPV = 93.7 % and NPV = 92.3 %), offered the best combination for predicting optimal cytoreduction. In addition, the HE4 value of 115 pmol/L is the best cut-off level for identifying platinum-sensitive patients.

**Conclusions:**

The introduction of HE4 as a new tool for predicting platinum-sensitivity and interval optimal cytoreduction is promising.

## Background

Epithelial ovarian cancer remains the main cause of mortality in patients with gynaecological malignancies. The annual incidence of ovarian cancer is 204,000; annualy, there are 125,000 deaths, and there is a close correlation between the stage at presentation and survival [1]. Cancer antigen 125 (CA125) is currently the only serological biomarker in routine use for managing patients with epithelial ovarian, fallopian tube and primary serous peritoneal cancer [2]. The upper limit of normal for CA125 is 35 UI/ml. Several studies have shown interest in using the CA125 value to predict optimal debulking, to evaluate platinum sensitivity and to monitor the disease after treatment [3].

Human epididymis protein 4 (HE4) is a novel and specific biomarker of ovarian cancer, and its expression is independent of CA125 [4]. The HE4 serum level in healthy women has been reported to range from 60 to 150 pmol/L. This wide range may be due to the relationship between increasing HE4 serum level and increasing age [5, 6]. Serum HE4 is more specific than CA125 in discriminating women with malignant tumors from those with benign tumours [7].

In addition to its diagnostic value, the serum HE4 level may be important for evaluating treatment response, predicting optimal cytoreduction and monitoring patients with ovarian cancer. Some teams have studied the evolving profile of the HE4 level during neoadjuvant chemotherapy (NAC) in small cohorts of patients deemed currently inoperable [8–10]. Moreover optimal tumor debulking and platinum response are the most important prognostic factors for overall survival in epithelial ovarian cancer (EOC) [11].

In this study, we aimed to analyse the predictive role of HE4 for surgical outcome and platinum response in advanced stage EOC patients deemed inoperable and to compare the results with those found for CA125.

## Methods

### Patients selection

Participants were recruited at two sites of Curie Institute (Paris and Saint Cloud, France). From January 2002 and December 2009, 117 patients with advanced epithelial ovarian cancer (FIGO stage III and IV) received NAC. Thirty patients had multiple sera collected during NAC and available for study. The inclusion criteria were the following: disease deemed inoperable and treated by neoadjuvant platinum-based chemotherapy and informed consent with agreement to undergo additional testing for new markers or additional histological explorations, even in hindsight. The protocol was reviewed and accepted by the Institutional Review Board. The patients underwent laparotomy or laparoscopy exploration with minimal surgery (biopsies), which was followed by NAC, interval debulking surgery and adjuvant chemotherapy. For each patient, the following clinical, biochemical, radiological and pathological variables were collected: age, weight, personal and family history, genetic predisposition, disease characteristics (histology, stage, and surgery) and relapse (treatment-free interval, location, and management).

### Measurement of CA125 and HE4

Venous blood samples were collected before chemotherapy treatment and interval debulking surgery. The B-R-A-H-M-S CA125 II Kryptor^R^ technique (Hennigsdorf, Germany), an automatic immunofluorescence analysis kit for measuring CA125 in the serum or plasma, was used to assay CA125. HE4 level was measured by a fully automated chemiluminescent Enzyme Immunoassay Lumipulse G HE4 (Fujirebio Europe, Gent, Belgium). These measurements were performed retrospectively from preserved samples. The threshold value for CA125 is commonly set < 35 UI/ml. The normal reference interval for HE4 is 32–108 pmol/L (2.5th percentile, 97.5th percentile) according the manufacturer.

### Statistical analysis

Statistical analyses were performed with R Version 3.2.2 software. The data are presented as the mean +/− standard derivation or median (range) and number (n). The Wilcoxon-Mann–Whitney test was used to analyze of quantitative variables, and the Fisher’s exact test was used for qualitative variables. We calculated the accuracy, sensitivity, specificity, positive and negative predictive value (PPV and NPV) of CA125 and HE4 alone and combined. Non-parametric receiver operating characteristic (ROC) analyses was performed to determine the optimal threshold of HE4 levels for predicting optimal surgery and platinum-sensitivity. To calculate the misclassification error rates we defined the best predictor using the Youden point on the ROC curve. The Youden index (YI) is defined as the maximum (sensitivity (YP) + specificity (YP) – 1), that occurs at the optimum threshold, the Youden point ((YP) [12]. We used the Optimal Cutpoints package. The diagnostic accuracy of the test was measured by the area under the curve (AUC). The bootstrap method was used to calculate 95 % confidence intervals. For the optimal threshold of the CA125 levels, we checked and used previously published thresholds. The overall survival was estimated using the Kaplan-Meier method and compared using the log-rank test. The platinum-free interval (PFI) is defined as the interval from the end of platinum-based chemotherapy to first recurrence. We chose the threshold value of 6 months to evaluate whether the disease was sensitive to platinum. The diagnosis of recurrence was based on clinical symptoms, clinically detectable disease and/or radiological evidence of disease recurrence. To assess the prognosis and peritoneal surface malignancy, we used the completeness of cytoreduction (CC) score, where CC-0 is defined as no residual macroscopic lesion after cytoreduction and CC-1, 2 and 3 (CC-1+) scores (tumour nodules persisting after cytoreduction less than 2.5 mm, between 2.5 mm and 2.5 cm, and greater than 2.5 cm or a confluence of unresected tumor nodules, respectively) were grouped together [13]. For all statistical comparisons, a *p*-value of < 0.05 was considered statistically significant.

## Results

**Fig. 1:**
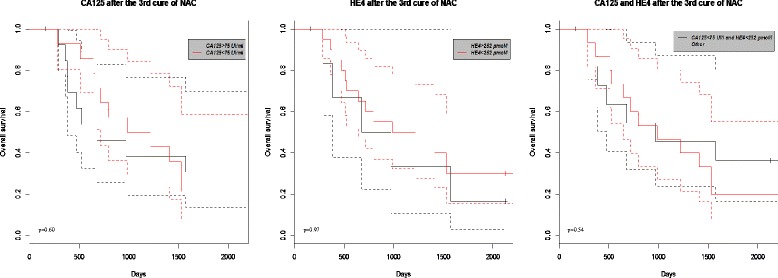
Kaplan-Meier estimates of the overall survival according to CA125 and HE4 cut-offs for predicting the optimal interval debulking surgery

The clinical characteristics and laboratory variables of the studied groups are reported in Table 1. The search for a genetic predisposition is made in accordance with age of patient, personal and family medical history. An oncogenetic consultation has been proposed for eight patients (less than one-third of our cohort). One mutated patient was found (BRCA1 mutation). Complete interval debulking surgery (IDS) was achieved in 16 of 30 (53 %) patients and was not complete in 14 of 30 (47 %) patients. The median age was 62.8 years (range 40–79). Ninety percent of patients were menopausal. All patients had serous adenocarcinoma and 50 % had grade 3 tumours according to final histology. All patients were eligible for NAC and had received taxane and platinum-based NAC. Building on our previous publications, we studied the tumour markers rates after the 3rd cycle of NAC [B]. The mean pre-NAC HE4 level was 928 pmol/L (range 46–6562 pmol/L) in the CC-0 group and 984 pmol/L (range 153–2746 pmol/L) in the CC-1+ group, and there was no significant difference. The mean pre-NAC CA125 level was 1432 UI/ml (range 16–9453 UI/ml) in the CC-0 group and 2257 UI/ml (range 632–9400 UI/ml) in the other. In our population, based on the ROC curve, the CA125 value of 75 UI/ml is the best cut-off to identify the patient candidates that are optimal cytoreduction agents with a sensitivity of 81.3 % and a specificity of 85.7 % (PPV = 86.7 % and NPV = 80 %). For CA125, the AUC is 0.92 (95 % confidence interval (CI) [0.80–1]). Instead, the cut-off level of HE4 with the best prognostic indices is 252 pmol/L, with a sensitivity of 93.3 %, a specificity of 50 % (PPV = 70 % and NPV = 85.7 %), and an AUC of 0.86 (95 % CI [0.68–1]). The CA125 and HE4 AUC indicate there is a good discrimination capability between the optimal and not optimal IDS cases. The cut-off for the combination of CA125 and HE4 considered in our study has a sensitivity of 93.7 % and a specificity of 92.3 % (PPV = 93.7 % and a NPV = 92.3 %). These results were significant and are summarized in Tables 2 and 3. In terms of survival, a trend towards improvement in overall survival was observed (Fig. 1) in the case of CA125, with HE4 decreasing below the thresholds above-mentioned.

**Table 1:** Patient characteristics

Characteristics	Overall population (*n* = 20)	CC-0 (*n* = 16)	CC-1+ (*n* = 14)	*p*-value	Relapse < 6 months (*n* = 12)	Relapse > 6 months (*n* = 18)	*p*-value
Age (years)	62.8 +/− 10.77	63.62 +/− 8.62	61.93 +/− 13.09	0.92	63.57 +/− 11.95	62.2 +/− 9.97	0.63
BMI (kg/m2)	23.64 +/− 3.39	23.69 +/− 2.91	23.59 +/− 3.98	0.77	22.75 +/− 3.04	24.42 +/− 3.58	0.24
Gestity	1.81 +/− 1.49	1.94 +/− 1.95	1.64 +/− 1.69	0.45	1.23 +/− 1.47	1.80 +/− 1.57	0.90
Parity	1.74 +/− 1.65	1.75 +/− 1.24	1.73 +/− 2.20	0.51	1.92 +/− 1.98	1.60 +/− 1.40	0.86
Menopause	27 (90 %)	15 (93.7 %)	12 (85.7 %)	0.59	10 (83.3 %)	17 (94.4 %)	0.59
FIGO stage				1			0.63
IIIa	1 (3.31 %)	1 (6.25 %)	0		0	1 (5.55 %)	
IIIb	1 (3.31 %)	1 (6.25 %)	0		0	1 (5.55 %)	
IIIc	23 (76.7 %)	12 (75 %)	11 (78.6 %)		9 (75 %)	14 (77.8 %)	
IV	5 (16.67 %)	2 (12.5 %)	3 (21.4 %)		3 (25 %)	2 (11.1 %)	
Grading				0.26			0.63
I	3 (10 %)	3 (18.7 %)	0		0	3 (16.7 %)	
II	9 (30 %)	5 (31.3 %)	4 (35.7 %)		5 (41.7 %)	4 (22.2 %)	
III	15 (50 %)	6 (37.5 %)	9 (64.3 %)		5 (41.7 %)	10 (55.6 %)	
Pre-NAC CA-125 (UI/ml)	1762.63 [16–9453]	1432.89 [16–9453]	2257.25 [302–9400]	0.05	1884.07 [16–9453]	1656.8 [57–9400]	0.15
Pre-NAC HE4 (pmol/l)	985.3 [46.1–6562]	928.22 [46.1–6562]	984.26 [153.2–2746]	0.24	1374.59 [63.9–6562]	644.66 [46.1–2031]	0.13
Cycles of NAC	5.75 [3–7]	5.5 [3–6]	6.12 [6–7]	0.08	5.60 [3–7]	5.62 [4–6]	0.69

*CC*-*0* non residual disease after interval debulking surgery (IDS), *CC*-*1*+ residual disease after IDS

*BMI* body mass index, *FIGO* International Federation of Gynecology and Obstetrics, *NAC* neoadjuvant chemotherapy

**Table 2:** Tumour markers after the 3rd cycle of NAC and the interval surgery outcome (a) or first relapse (b)

(a)			
	CC-0 (*n* = 16)	CC-1+ (*n* = 14)	*p*-value
CA125 ≤ 75 UI/ml	13 (81.3 %)	2 (12.3 %)	0.0007
HE4 ≤ 252 pmol/l	14 (87.5 %)	6 (42.8 %)	0.02
CA125 ≤ 75 UI/ml and HE4 ≤ 252 pmol/l	15 (93.7 %)	1 (7.1 %)	0.00001
(b)			
	Relapse < 6 months (*n* = 12)	Relape > 6 months (*n* = 18)	*p*-value
CA125 ≤ 35 UI/ml	1 (8.3 %)	10 (55.5 %)	0.018
HE4 ≤ 115 pmol/l	1 (8.3 %)	13 (72.2 %)	0.0017
CA125 ≤ 35 UI/ml and HE4 ≤ 115 pmol/l	1 (8.3 %)	13 (72.2 %)	0.0017

**Table 3:** Performance tumour markers in predicting cytoreduction (a) or platinum sensitivity (b)

	Sensitivity	Specificity	PPV	NPV	DOR
(a)					
CA125 ≤ 75 UI/ml	81.3 %	85.7 %	86.7 %	80 %	22.13
HE4 ≤ 252 pmol/l	93.3 %	50 %	70 %	85.7 %	12.57
CA125 ≤ 75 UI/ml and HE4 ≤ 252 pmol/l	93.7 %	92.3 %	93.7 %	92.3 %	96.15
(b)					
CA125 ≤ 35 UI/ml	90.9 %	57.9 %	55.6 %	91.7 %	13.75
HE4 ≤ 115 pmol/l	92.9 %	68.7 %	72.2 %	91.7 %	28.6
CA125 ≤ 35 UI/ml and HE4 ≤ 115 pmol/l	92.9 %	68.7 %	72.2 %	91.7 %	28.6

*PPV* positive predictive value, *NPV* negative predictive value, *DOR* diagnostic odd ratio

In our cohort, 12 patients (40 %) experienced recurrence in the first 6 months, and 18 patients (60 %) were considered platinum-resistant with a first relapse after 6 months. The clinical and biological characteristics were similar in both groups (Table 1). The average value of pre-NAC HE4 and CA125 were similar in both groups and were statistically not significant. In 30 women with EOC, HE4 appear to improve the prediction of the platinum sensitivity (Table 2). Based on ROC analysis, the CA125 and HE4 cut-off values for predicting platinum sensitivity were 35 UI/ml and 115 pmol/L, respectively, and they were accompanied by AUC values of f 0.80 (95 % CI [0.62–0.94]) and 0.88 (95 % CI [0.73–0.98]), respectively, for CA125 and HE4. At the ideal cut-off, corresponding to the highest accuracy (minimal false-negative and false-positive results), HE4 and the combination CA125 + HE4 resulted in a similar sensitivity, specificity, PPV and NPV (Table 3).

## Discussion

Ovarian cancer is usually diagnosed at an advanced stage. In the advanced stage, many patients have multiple peritoneal locations, making it difficult to completely debulk the tumours in these patients. Based on numerous recent studies, NAC appears to be a valuable option for patients who cannot undergo surgery with optimal cytoreduction performed on them [14, 15]. An optimal surgical outcome is one of the most powerful determinants of survival [13]. NAC, followed by surgical debulking, can achieve survival rates that are equivalent to that observed with primary surgical debulking followed by adjuvant chemotherapy [14]. First-line of chemotherapy is based on platinum in ovarian cancer. The platinum response is an independent prognostic factor for the overall and progression-free survival in patients with EOC [16]. Unfortunately, there has been no general consensus on the best preoperative approach to predict cytoreductibility. Similarly, it is difficult to predict the platinum sensitivity of the disease before the first relapse. Several studies have evaluated the role of CA125 in predicting cytoreductibility. For patients receiving primary cytoreduction, a preoperative CA125 level of 500 UI/ml was used as the proper cut-off limit for this purpose [17–22]. For patients receiving NAC, different cut-offs were published, ranging from 20 to 100 UI/ml [23–25]. Our result indicates that a CA125 level after the 3rd cycle of NAC of 75 UI/ml could help identify patients in whom optimal cytoreduction will be achieved. The same cut-off was published by Braicu et al. [26]. HE4 is a new tumour marker that was recently approved for diagnosing and monitoring ovarian cancer [5]. Data on the role of HE4 in the carcinogenesis are inconsistent. There are a few small studies evaluating the profile of HE4 during NAC in a primarily inoperable ovarian cancer patient cohort [8–10]. A study of 10 patients showed that the profile of HE4 during NAC was in line with radiologic and clinical responses. In the NAC group, HE4 correlated better with the radiologic response than CA125 [10]. A Yang et al. showed that 600 pmol/L is the cut-off value for HE4, above that level cytoreductive surgery should be deferred and the sensitivity and specificity of the test were 77 and 32 %, respectively [27]. In our study, the cut-off value for HE4 was lower; calculated based on the method by Youden,it was 255 pmol/L resulting in a sensitivity and specificity of 91.7 and 67 %, respectively. Angioli et al. (262 pmol/L), Chudecha-Glaz et al. (218.43 poml/L) and Braicu et al. (250 pmol/L) presented HE4 cut-off values that were similar to ours [26, 28, 29]. At the same time, they showed that HE4 is a better predictor than CA125 of the feasibility of optimal cytoreduction. In our study, HE4 and CA125 show similar performance in predicting surgical cytoreduction with diagnostic odds ratios (DOR) of 21.97 and 21.00 respectively. In combination, the diagnostic accuracy is strongly enhanced with a DOR above 500 (Table 3). The best factor in predicting cytoreduction was the combination of CA125 ≤ 75 UI/ml and HE4 ≤ 252 pmol/L, which had a sensitivity of 93.7 % and specificity of 92.3 % (PPV = 93.7 % and NPV = 92.3 %). Angioli et al. showed similar results with a sensitivity of 88.8 % and specificity of 89.5 % (PPV = 94 % and NPV = 80 %) for the combination CA125 (≤414 UI/ml) + HE4 (≤262 poml/L). However, in this publication, the best association in predicting primary cytoreduction is HE4 level ≤ 262 pmol/L and ascites ≤ 500 ml (sensitivity = 100 %, specificity = 89.5 %, and *n* = 36 patients). To the best of our knowledge, no publication is available on the ability of HE4 to predict platinum sensitivity. Unfortunately, it is difficult to compare our data. The benefit of HE4 for predicting platinum sensitivity seems limited. Larger population studies are needed to evaluate these data.

The limitations of our study include its retrospective nature and probable selection bias. In addition, the HE4 levels were not available for some patients, limiting the number of patients whose data could be analyzed and the statistical power of our analysis. This is a pilot study and larger studies are needed.

## Conclusions

In conclusion, CA125 is still the only tumour marker that is recommended as a diagnostic or prognostic indicator and for monitoring disease recurrence after surgery and chemotherapy [30]. HE4 had comparable diagnostic performance with CA125 as a tumour marker for detecting ovarian cancer. HE4 was more sensitive and specific in detecting the early stages of ovarian cancer and more specific [30]. Based on our results and the literature, the introduction of HE4, alone or combined with CA125, as a new tool for predicting platinum-sensitivity and primary or interval optimal cytoreduction is promising.

## Abbreviations

AUC: Area under the curve
BMI: Body mass index
CC score: Cytoreduction score
CC-0: Non residual disease
CC-1+: Residual disease
DOR: Diagnostic odd ratio
FIGO: International Federation of Gynecology and Obstetrics
IDS: Interval debulking surgery
NAC: Neoadjuvant chemotherapy
NPV: Negative predictive value
PFI: Platinum free interval
PPV: Positive predictive value
ROC: Receiving operating characteristic
YI: Youden index
YP: Youden point
